# Zika virus proteins at an atomic scale: how does structural biology help us to understand and develop vaccines and drugs against Zika virus infection?

**DOI:** 10.1590/1678-9199-JVATITD-2019-0013

**Published:** 2019-08-29

**Authors:** Ana Paula Valente, Adolfo Henrique Moraes

**Affiliations:** 1 National Center of Magnetic Resonance, Leopoldo de Meis Institute of Medical Biochemistry, Federal University of Rio de Janeiro (UFRJ), Rio de Janeiro, RJ, Brazil.; 2 Department of Chemistry, Institute of Exact Sciences, Federal University of Minas Gerais (UFMG), Belo Horizonte, MG, Brazil.

**Keywords:** Zika virus proteins, structural biology, protein structure, nuclear magnetic resonance spectroscopy, protein-antibody interaction, protein-small compound interaction

## Abstract

In Brazil and in other tropical areas Zika virus infection was directly associated with clinical complications as microcephaly in newborn children whose mothers were infected during pregnancy and the Guillain-Barré syndrome in adults. Recently, research has been focused on developing new vaccines and drug candidates against Zika virus infection since none of those are available. In order to contribute to vaccine and drug development efforts, it becomes important the understanding of the molecular basis of the Zika virus recognition, infection and blockade. To this purpose, it is essential the structural determination of the Zika virus proteins. The genome sequencing of the Zika virus identified ten proteins, being three structural (protein E, protein C and protein prM) and seven non-structural proteins (NS1, NS2A, NS2B, NS3, NS4A, NS4B and NS5). Together, these proteins are the main targets for drugs and antibody recognition. Here we examine new discoveries on high-resolution structural biology of Zika virus, observing the interactions and functions of its proteins identified via state-of-art structural methodologies as X-ray crystallography, nuclear magnetic resonance spectroscopy and cryogenic electronic microscopy. The aim of the present study is to contribute to the understanding of the structural basis of Zika virus infection at an atomic level and to point out similarities and differences to others flaviviruses.

## Background

The Zika virus (ZIKV) has become one of the major threats to public health systems worldwide. This virus was first isolated in 1947 from a sentinel rhesus monkey at the Zika forest, in Uganda [1]. ZIKV is a member of the *Flavivirus* genus, together with dengue virus (DENV), yellow fever virus (YFV) and West Nile virus (WNV). Like its relatives, ZIKV is transmitted to humans through the bite of infected *Aedes* mosquitoes. ZIKV can also be transmitted from an infected pregnant woman to her fetus during gestation leading to severe birth defects as congenital microcephaly [2]. Other forms of transmission have also been described, including sexual and blood-borne [3,4]. 

In May 2015, the Pan American Health Organization issued an alert in response to the first confirmed ZIKV infection in Brazil. In November 2015, the first microcephaly case potentially related to the ZIKV infection was reported. Since then, the scientific community has joined efforts to accelerate the development of new vaccines and antivirals against ZIKV. Government, academia, pharmaceutical and biotech industries are focusing in the understanding of the disease and its causes to develop effective strategies of combating it. For those purposes, it becomes crucial the detailed description of the molecular basis of the ZIKV recognition, infection and blockade. High-resolution structural knowledge allows us to understand and identify exposed epitopes and druggable sites, crucial steps to design effective vaccines and structure-based drugs against ZIKV [5,6]. 

ZIKV is a positive single-stranded RNA virus with a 10.7 kb genome translated into a single polyprotein of about 3.000 amino acids. During the viral replication, the polyprotein is cleaved to produce three structural proteins involved in the viral particle assembly, namely the glycoprotein E (protein E), the capsid protein C (protein C), and the protein prM. Whereas seven non-structural proteins are responsible for the viral replication, assembly and evasion from the host defense: NS1, NS2A, NS2B, NS3, NS4A, NS4B and NS5 [1]. These proteins are the main targets for structure-based antiviral discovery and antigen identification for vaccine development [1,7].

As ZIKV spread around the world, the structural biology community has been combining efforts to obtain high-resolution structures of ZIKV proteins by using state-of-art methodologies such as X-ray crystallography, biomolecular nuclear magnetic resonance (NMR) spectroscopy and cryogenic electronic microscopy (CryoEM). In about one year, those efforts provided important structural information on ZIKV proteins. Structures of six out of ten proteins of ZIKV were already solved by X-ray crystallography: protein E, protein C, NS3 (helicase domain and protease domain), NS2B, NS5, and NS1 (Table 1). In the present review, the most important results recently obtained on the structure biology of Zika virus are summarized. In the following sections, we are going to list the main conclusions that could be drawn from those structures and compare them to similar proteins from other flaviviruses. 

**Table 1.** Structural information on Zika virus proteins

Protein	PDB	Ligand	Reference
Glycoprotein E	5KVE-DIII	ZV-48 Antibody scFv	[8]
	5KVD-DIII	ZV-2 Antibody Fab	[8]
	5KVF-DIII	ZV-64 Antibody Fab	[8]
	5KVG-DIII	ZV-67 Antibody Fab	[8]
	5LBS	Human antibody EDE1 C8 - scfv	[9]
	5LBV	-	[9]
	5LCV	Human antibody EDE2 A11-FAB	[9]
	5JHL	FAB	[10]
	5JHM	-	[10]
NS3 helicase	5JWH	-	[11]
	5K8I	ATP	[11]
	5K8L	GSP	[11]
	5K8T	GSP, Mg^2+^	[11]
	5K8U	ADP, Mn^2+^	[11]
	5GJB	ss-RNA	[12]
	5GJC	ATP, Mn^2+^	[12]
	5JRC	-	[13]
	5JMT	-	[14]
NS2B-NS3 serine protease	5GJ4	Last four amino acids of NS2B cofactor	[15]
	5T1V	-	
	5LCO	Boronate inhibitor	[16]
NS5 methyltransferase	5TFR	2 Zn^2+^	[17]
	5TMH	2 Zn^2+^, S-adenosyl-L-homocysteine	
	5U0B	2 Zn^2+^, S-adenosyl-L-homocysteine	[18]
	5U0C	2 Zn^2+^	[18]
	5KQR (MTase)	S-adenosylmethionine	[19]
	5KQS (MTase)	S-adenosylmethionine,7-methyl-guanosine-5'-diphosphate	[19]
NS1	5K6K	-	[20]
	5IY3	-	[21]
	5GS6	-	[22]

## Near-atomic resolution structure of Zika virus

X-ray crystallography was the first technique used to solve atomic-resolution structures of viruses during 1970s and 1980s [23,24]. Since then, hundreds of atomic-resolution structures were obtained. According to VIPERdb database of icosahedral virus capsid structures (http://viperdb.scripps.edu) about 400 virus structures have been solved by X-ray crystallography. However, this experimental approach has some limitations, since it remains difficult to achieve atomic resolution for large viruses as the flaviviruses [25]. Therefore, up to this moment, no flavivirus atomic-resolution structure has been solved by X-ray crystallography. 

Single-Particle CryoEM is nowadays one of the main tools used to obtain near-atomic resolution structures (2-4 Å) of isolated viruses. Since 2008, when a resolution of about 4.0 Å was reached [26,27,28], many viruses have had their structures solved by CryoEM, including Zika [25,29,30]. 

**Figure 1.**
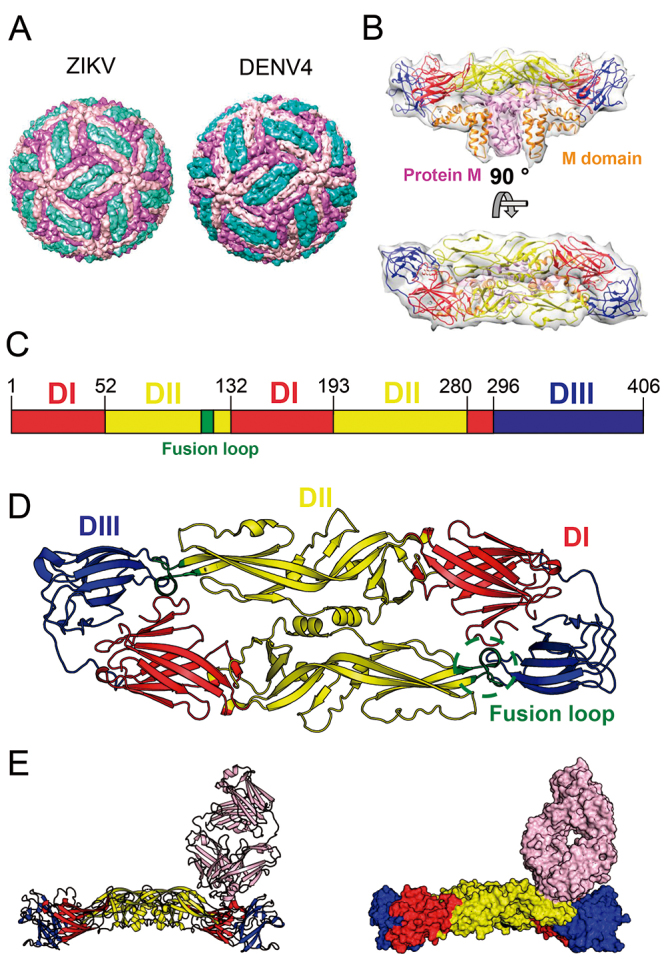
(A) CryoEM structure of ZIKV (PDB ID: 5IZ7) and DENV4 (PDB ID: 4CBF). (B) Model of protein E and protein M based on Cryo-EM density maps (PDB ID: 5IZ7). (C) Schematic domain organization of the ectodomain of ZIKV protein E. (D) Structure of ZIKV protein E dimer (PDB ID: 5JHM) and its three domains. (E) Structure of ZIKV-antibody complex (PDB ID: 5LCV).

Previous studies have shown that the ZIKV surface shares similar structure and composition to other flaviviruses like DENV and WNV (Figure 1A and B). They have 180 copies of the protein E, associated with protein M. Protein E copies can assemble into an icosahedral symmetry formed by 90 dimers at pH around 7.4. This complex structure is anchored in the lipid bilayer by their intermembrane domains [30,31,32,33,34]. 

In spite of sharing the same general structure to other flaviviruses, ZIKV has some unique physical-chemical properties: it is more stable than the others, as DENV, for instance; its surface is smoother than those of other flaviviruses even at 40 °C; when it is still infective [33,34]. Its optimal thermal stability and infectivity at higher temperatures could be associated with the resistance of ZIKV particle in a variety of environments as those found in urine, semen, breast milk, and saliva [35,36,37,38]. 

Despite all the results that CryoEM has been providing, some limitations to near-atomic resolution of flaviviruses structure yet persist. One example is the lack of structural information of flavivirus nucleocapsids, formed by protein C. This happens because the nucleocapsid symmetry is not icosahedral [39]. Another important needed development is the addition of the conformational dynamics contribution to the structure of viruses, since many virus proteins undergo dramatic conformational changes during host cell recognition and infection, assembly of virus particle and viral releasing from host cell. The flavivirus protein E, for instance, morphs from dimer to trimer conformations, which is triggered by pH variation during the virus and host cell endosomal membrane fusion and during viral host-releasing [40,41]. 

## Protein E: the major component of flavivirus surface

Protein E is the major component of flavivirus surface. This protein is involved in host cell receptors recognition, virus entry process and viral assembly [42]. It is worth noticing that protein E conformational changes induced by pH variation, from extracellular environment to endosomal ones, mediates the virus-host cell endosomal membrane fusion. It also has an important role in the immune response, representing the major target of neutralizing antibodies [9,10].

ZIKV protein E dimer structures (PDB ID: 5LBV and 5JHM) show that each protein E is composed by three domains: domain I (DI), domain II (DII) and domain III (DIII) (Figure 1C and D). DI is a non-continuous β-shaped domain [10,41], which is responsible for linking DII to DIII. It acts on fundamental conformation changes in protein E during flavivirus infection [41]. DII is a non-continuous finger-like domain. Many DII residues participate on the hydrogen and electrostatic interaction net stabilizing the protein E dimers. The fusion peptide is also located in the DII and has a conserved amino-acid sequence that interacts with host cell endosomal membrane during the virus-host cell membrane fusion process [10]. The C-terminal immunoglobulin-like DIII has high homology to DENV. The interaction between DIII and some glycosaminoglycans is associated with the primary interaction between the viruses and host cells [42,43,44,45,46]. Among all solved structures for protein E, dengue virus serotype 3 (DENV3), has the higher sequence identity to ZIKV (sequence identity of 56%) and, DENV 2 has the most similar dimer structure (Dali Z factor 54.6 and RMSD 2.6).

As mentioned before, the protein E is the main target for immune response against flaviviruses, which is not different for ZIKV. Therefore, the structural and conformational change characterization of protein E and its interaction with different antibodies is necessary for ZIKV vaccine development or even to new passive immunization strategies based on optimized antibodies or antibody fragments, as the fragment antigen-binding (Fab), and the single-chain variable fragment, scFv. For example, some structures of complexes of protein E and neutralized antibodies have been obtained by X-ray crystallography and CryoEM at atomic and near-atomic resolution (Figure 1E) [8,10,47]

Nowadays, NMR spectroscopy emerges as an important technique to identify protein conformational epitopes in solution. NMR is an ideal methodology that describes at the atomic level the antigen-antibody complex in solution. Bardelli *et al.* [48] described the characterization of epitopes by NMR, showing its advantages and drawbacks. The practical approaches include labeling the antigen (^15^N-^13^C) by using heterologous expression systems. Antibodies used in those assays can be isolated from patients or animal models or even be recombinant antibodies or antibody fragments (Fab and scFv). Titration NMR experiments with antibodies allow the monitoring of the chemical environment changes of amide groups by ^1^H-^15^N NMR correlation experiments. Those amide groups in which chemical environment was altered upon titration are strong candidates to be located on one antigen conformational epitope. Together with computational methodologies as molecular docking, NMR results provide structural models for antigen-antibody complexes in a fast way [49,50,51]. This methodology has some limitations, though. The main one is the molecular size of the complex that cannot exceed 100 kDa. This problem can be overcome by using recombinant DIII of protein E with antibody fragments Fab and ScFv, that have lower molecular weight than a whole antibody [48,52]. Another way to study these complexes is by measuring the molecular dynamics of ^15^N nucleus of antigens upon titration with antibodies by relaxation NMR experiments [52].

## NS1

NS1 is a ~48 kDa protein important for genome replication, pathogenesis, and host immune response modulation [20]. In other flaviviruses, NS1 is a glycosylated homodimer localized inside the host cell, more specifically in the endoplasm reticulum (ER) lumen. It participates in the replication complex together with the transmembrane proteins (NS2A, NS4A and NS4B) and with the cytoplasmic proteins NS3 helicase, NS2B co-factor and NS5. NS1 is also secreted from host cells as a lipoprotein hexamer to extracellular medium, where it modulates the host immune response and pathogenesis by interacting with host factors and components of the innate and adaptive immune systems [53]. NS1 also interacts with other flavivirus proteins like proteins prM and protein E [54]. 

**Figure 2.**
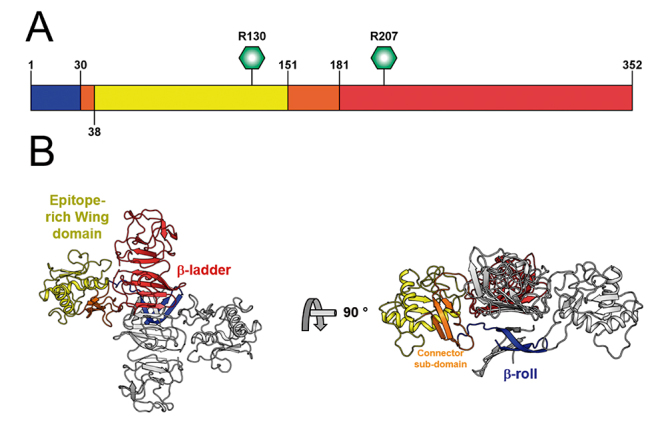
(A) Domain organization of NS1. Glycosylated residues R130 and R205 are identified by hexagons. (B) X-ray crystal structure of ZIKV NS1 dimmer (PDB ID:5GS6). ZIKV domains are colored following the color pattern of panel A.

Solved structures of ZIKV NS1 showed three domains: an N-terminal β-roll, an epitope-rich ring, and a C-terminal β-ladder. These domains are colored in Figure 2A and B in blue, yellow, and red, respectively. The NS1 homodimer has a cross-shaped fundamental unit stabilized via intertwined β-roll and end-to-end ladders interactions. The membrane interaction occurs via a hydrophobic core on the inner face of the homodimer formed by the β-roll domains and by the “greasy finger”. The outer face of NS1 is polar with specific sites for glycosylation. Residues 290, 293, 297, 300, 301 and 304 have been identified as O-glycosylation sites [20,21].

Two regions of NS1 dimer have been associated with ER interaction: the hydrophobic region of the connector sub-domain and the wing-flexible loop of the wing domain. Brown *et al.* [20] characterized the interaction of NS1 with liposomes, showing the conversion of heterogeneous large liposomes into a much smaller lipid-protein nanoparticles.

During translation, NS1 is moved to the lumen of ER and the N-term is cleaved by a host cell peptidase, followed by NS1-NS2A junction cleavage by an unknown protein at the position L/M-V-X-S-X-V-X-A, which is a good target for inhibitor development [55]. The hydrophobic core on the inner face of the homodimer formed by the β-roll domains and by the “greasy finger” is another good target, due to its ability to mediate NS1-host cell lipid interaction. Gutsche *et al.* [53] characterized the hexamer structure of DENV, revealing the presence of triglycerides, cholesteryl esters and phospholipids in this complex.

## NS2B-NS3

**Figure 3.**
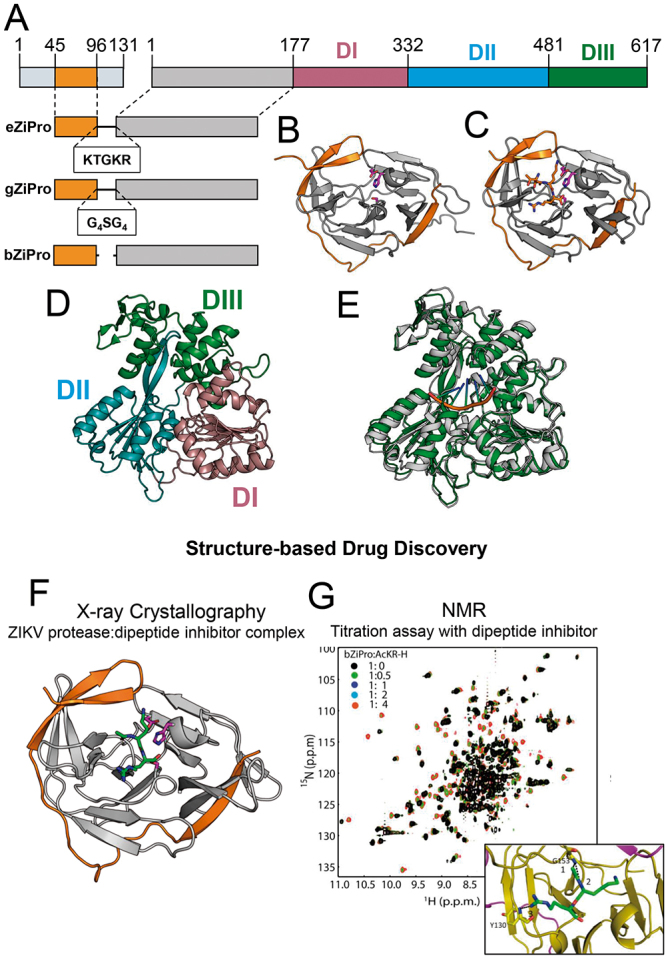
(A) Schematic domain organization of ZIKV NS3. (B), (C) Unlinked NS2B-NS3 protease complex (bZipPro) in close conformation (PDB ID: 5GPI) and NS2B-NS3 protease complex (eZipPro) after cleavage also in close conformation (PDB ID: 5GJ4), respectively. Side chains of catalytic residues H51, D75 and S135 are colored in magenta. (C) The processed tetrapeptide TGKR from the linker region is colored in orange. (D) Subdomain organization of NS3 helicase (PDB ID: 5JMT). (E) Superposition of crystal structures of NS3 helicase free (grey) (PDB ID: 5JMT) and complexed with single-chain RNA fragment (AGAUC) (green) (PDB ID: 5GJB). (F) Crystal structure of NS2B-NS3 protease-dipeptide inhibitor complex (PDB ID: 5MRK). (G) ^1^H-^15^N HSQC spectra of NS2B-NS3 protease bZiPro in the absence and presence of dipeptide inhibitor AcKR-H and structure of the bZiPro- AcKR-H complex adapted from [56].

NS3 is one of the components of the ZIKV replication complex. NS3 is anchored in the ER by strong interactions of its N-terminal with the hydrophilic region of integral membrane protein NS2B. The NS3 C-terminal domain possesses helicase and NTPase activities and is known as helicase domain. The N-terminal together with its cofactor, NS2B, forms a protease involved in the cleavage of the flavivirus polyprotein (Figure 3). Because of its multiple roles in the virus cycle, N2B-NS3 protein is one of the main targets for drug screening against flaviviruses. The structure of the entire ZIKV NS2B-NS3 protein was not solved, but a variety of structures of the NS3 helicase and NS2B-NS3 protease domains were obtained through X-ray crystallography. Many of them, with natural substrates and/or drug candidates bound to one of the domains. In the following sections, we are going to describe the major contributions that structure biology has provided to a better understanding of the properties and functions of both domains of NS2B-NS3 protease.

## NS3 helicase domain

The ZIKV helicase/NTPase domain, ZIKV NS3 helicase, belongs to the helicase superfamily 2 (SF2) and is responsible for the unwinding of structured template regions of RNA, working in a coordinated way with NS5 polymerase during the *de novo* viral RNA synthesis. NS3 helicase has also nucleoside triphosphatase activity, which is stimulated by RNA binding and provides the chemical energy necessary for unwind RNA intermediates during viral RNA replication [57]. There is also strong indication that in the flavivirus replication complex, NS3 interacts with NS5 polymerase. Tay *et al.* [58] used competitive interaction Elisa and Small Angle X-ray Scattering (SAXS) to show that ~50 residues of DENV NS3 are participating of NS3 and NS5 interaction.

X-ray crystallography has vastly contributed to the understanding of ZIKV NS3 helicase function and structural properties. Many high-resolution flavivirus NS3 helicase structures have been obtained for ZIKV, DENV2, DENV4, JEV, KUNV, YFV and MVEV [12,56,59,60,61,62,64]. Among them, DENV2 NS3 helicase is the closest one to ZIKV, either regarding primary sequence identity (72%) or tertiary and quaternary structure (Z factor and RMSD of 54.6 and 1.3, respectively). Structure-based phylogenetic tree showed that ZIKV NS3 helicase can be clustered into group 1a with DENV2, DENV4 and MVEV. NS3 helicase from YFV, KUNV and JEV form the group 1b and HCV forms a separated group 2 [14]. ZIKV NS3 helicase is monomeric in solution and its structure is composed of three domains: domain I, DI (residues 175-332), domain II, DII (residues 333-481), and domain III, DIII (residues 482-617) [12,14]. The structure and schematic domains are shown in Figure 3A, D and E.

Eight different structural motifs involved in RNA binding, ATP hydrolysis and communications between the binding sites are located in the ZIKV NS3 helicase domain [12,57,60]. Residues involved in catalytic activities are conserved among other flaviviruses, indicating that functions are also preserved, which was corroborated by Tian *et al.* [14], who obtained the crystal structure of ZIKV NS3 helicase domain. NS3 helicase DI and DII share a similar RecA-like fold but have low sequence identity. Both are composed by a β-sheet formed by six β-strands stacked between loops and four α-helices. DIII is composed by four α-helices and two β-strands. Some important information regarding the dynamics of NS3 helicase can be observed from crystal structure B-factor. Amino acid residues 193-202 and 249-255 showed higher B-factor values indicating higher flexibility. Another important information is that priming loop adopts different conformations in different flaviviruses but, apparently, a similar conformation when ATP/Mg2+ is bound. The ss-RNA site is in a cleft formed by positive residues in DI and DII [12].

The high-resolution structures of ZIKV NS3 helicase [12,14] together with a diversity of structures of other flaviviruses provide an outstanding help for structure-based drug discovery of flavivirus NS3 inhibitors. The ZIKV NS3 substrate-binding sites are good targets for substrate-analogue NS3 inhibitor candidates. In this regard, the structural characterization of the ATP/Mn^2+^ and RNA binding to the flavivirus helicase carried out by Tian *et al.* [12] is especially important for NS3 inhibitor design. The ATP/Mn^2+^ binding site is in a cleft of DI and DII, and is composed by residues G197, K200, T201, R202 (P loop), E286, N330, R459 and R462. The ss-RNA binding site is located in a tunnel that goes from DII to DI opposed by DIII and is consistently positively charged. Due to the similarity of the substrate-binding sites of flavivirus NS3 helicase with those from human helicase/ATPase proteins, no NS3 helicase substrate-analogue inhibitor candidate was clinically approved. However, other sites in NS3, mainly those related to allosteric events, could be good targets for inhibitor discovery. In this case, NMR Spectroscopy can be of great help. 

## NS2B-NS3 serine protease

The NS2B-NS3 serine protease is essential for understanding the flavivirus cycle within a host cell. Together with host cell proteases, NS2B-NS3 processes the synthesized polyprotein, thus being indirectly responsible for virus assembly and replication. NS2B protein has a transmembrane region attached to the ER lipid membrane and a hydrophilic region that interacts with the N-terminal of NS3 protein (Figure 3B and C), acting as its cofactor. NS2B-NS3 protease is responsible for all cytoplasmic cleavages at junctions between NS2A/NS2B, NS2B/NS3, NS3/NS4A and NS4B/NS5 proteins and within the capsid, NS2A and NS4A proteins. In other flaviviruses, NS2B-NS3 also plays several roles as being the basis for the formation of virus replication complex, interacting with transmembrane protein NS4B and NS5 polymerase, and even modulating the pathogenesis and host immune cell response [16,56,57,65]. The catalytic site is in the N-terminal of NS3 and is basically formed by the triad of residues S165, H51 and D75 [15,66,67]. 

Structure characterization of NS2B-NS3 protease have evolved after Leung *et al.* [66] engineered a dengue virus NS2B-NS3 synthetic fusion construct that remedied the lack of solubility of NS2B-NS3 protease. These and other studies were the basis for the structure determination of ZIKV NS2B-NS3 protease and of other flaviviruses. Some constructs in which hydrophobic regions of NS2B and NS3 are deleted have been crystalized and displayed strong peptidolytic activity [66]. The organization of three from the most popular constructs of NS2B-NS3 - eZiPro, bZiPro, and gZiPro - are shown in Figure 3A. The three crystalized constructs stabilized in the closed conformation state, even in the absence of a substrate or inhibitor. The conservation of the closed conformation state could be an important difference between ZIKV NS2-NS3 and DENV protease, since in DENV the apo protease is in an opened conformation, characterized by the unfolding of NS2B cofactor. The conformational state in DENV protease is only stabilized when the protein complex is bound to a ligand [63]. From these studies, it was possible to characterize protease catalytic activity of different constructs and to obtain valuable information for experimental screening assays. 

Many pockets involved in the substrate binding and suitable for substrate-analogue inhibitor candidate design were identified from the crystal structures of flavivirus NS2B-NS3 proteases. Two good examples of that are hydrophobic S2 and S3 pockets formed by the NS2B β-hairpin (residues 68-99) and the substrate-binding site of NS3 protease domain [15,57]. One example of the application of X-ray crystallography in the search for an inhibitor of NS2B-NS3 protease was given by Lei *et al.* [16], that obtained the NS2B-NS3 protease bound to a peptidomimetic boronic acidic inhibitor (cn-716) .

NMR spectroscopy has also being used and have provided new information on NS2B-NS3 protease structure, dynamics and activity. Using NMR ^15^N-relaxation experiments Zhang *et al.* [65] mapped the molecular dynamic of the unbound and bound states of bZiPro construct. Unbound and bound ZIKV NS2-NS3 showed similar dynamics, confirming that both are in closed conformation. Residues located in the catalytic site presented line broadening, possibly due to conformation exchange in the unbound state. Another example that shows the contribution of NMR to the NS2B-NS3 protease structure-based drug discovery efforts was provided by Mahawaththa *et al*. [68], who characterized the conformational equilibrium between the closed and opened conformations of NS2B-NS3 protease free and bound to three different inhibitors. 

To exemplify the potential of combined approaches on the ZIKV NS2B-NS3 protease inhibitor design, we can mention a study carried out by Yan Li *et al.* [56]. They solved the structure of the protease in complex with Acyl-KR-aldehyde, a dipeptide inhibitor, using X-ray crystallography (Figure 3F) and characterized the conformational equilibrium of the complex by using NMR spectroscopy. Moreover, they proved that NMR could be used as a high-resolution and fast-carrying technique for experimental screening of NS2B-NS3 inhibitor candidates, validating their strategy with two other compounds (Acyl-K-Agmatine and Acyl-KR-COOH) (Figure 3G). 

## NS5 protein

**Figure 4.**
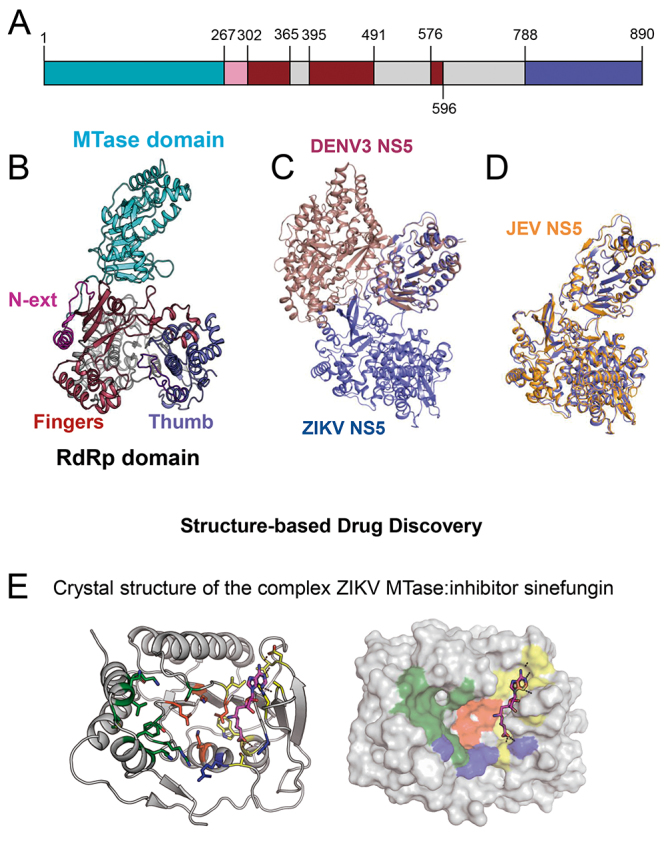
(A) Schematic domain organization of ZIKV NS5. (B) Structure of ZIKV NS5 (PDB ID: 5FTR). (C) Superposition of NS5 structures of DENV3 (PDB ID: 5CCV) and ZIKV (PDB ID: 5FTR). (D) Superposition of NS5 structures of JEV (PDB ID: 4K6M) and ZIKV (PDB ID: 5FTR). (E) Structure of NS5 Mtase complexed with SAM-analogue inhibitor sinefungin (PDB ID: 5MRK).

NS5 is part of flavivirus RNA replication complex. NS5 is the largest protein encoded in the flavivirus genome and is the most conserved one among viral proteins. This protein plays a crucial role in flavivirus replication and is an important target for drug development. Structure of full NS5 proteins of ZIKV, DENV 3, and JEV were solved by X-ray crystallography [18,69,70,71]. Based on structural data and on primary sequence analysis, the structure of NS5 can be decomposed in two structural and functional domains: a RNA methyltransferase domain (MTase domain) in the N-terminal and a RNA-depended RNA polymerase domain (RdRp domain) in the C-terminal (Figure 4A and B). ZIKV NS5 has 70% of primary sequence identity and possesses a very similar tertiary structure to JEV NS5. Residues 263-273 are poorly conserved across flaviviruses and form a linker between the catalytic domains of NS5. The alignment of MTase domain (residues 1-263) of ZIKV and DENV3 NS5 showed rms value of 0.664 Å and 0.593 Å with JEV NS5. Using the RdRp domain (residues 273-896) the rms was 1.120 Å with DENV3 and 0.525 Å with JEV (Figure 4C and D). 

### NS5 MTase domain

The ZIKV NS5 MTase domain (residues 1-263) is involved in the viral mRNA cap formation, more precisely, it methylates the mRNA cap in different positions, what is essential in viral replication and for avoiding virus detection by innate immune mechanisms [72]. During mRNA methylation, flavivirus MTase binds to ss-mRNA and to the methyl donor S-adenosyl-L-methionine (SAM). As NS5 MTase domain displays such important function in flavivirus replication, it becomes, along with NS5 RdRp domain and NS2B-NS3 protease, an important target for drug discovery against flaviviruses [73]. Many high-resolution structures of MTase domain from ZIKV NS5 protein free or complexed with different substrates, cofactors and substrate-analog molecules have been solved by X-ray crystallography [17,19,74,75]. As other flavivirus MTases, ZIKV MTase has a Rossmann fold with seven β-strands surrounded by four α-helices. The sequence identities between the ZIKV NS5 MTase and those from WNV, JEV, MVEV, DENV and YFV are 70%, 70%, 69%, 61% and 55%, respectively. The superposition of ZIKA NS5 structures bound to guanosine triphosphate, GTP, S-Adenosyl-L-homocysteine, SAH, and those of other flaviviruses showed nearly identical structures [17]. As the flavivirus MTases present high sequence identity, structure and high conservation of residues in the binding sites of mRNA and SAM, it is likely that a given inhibitor, developed for one virus, could be effective against a variety of flavivirus MTases. 

The search for MTase inhibitors is generically focused on substrate-analogue candidates. Therefore, X-ray crystallographic-derived high-resolution structures are fundamental to this endeavor. The description of the mRNA and SAM binding sites, which are highly conserved among flavivirus MTases, is especially relevant. It is worthwhile mentioning four sites or residue clusters in ZIKV MTase involved in substrate binding:

- mRNA cap-binding site: K13, L16, N17, M19, F24, K28, S150, R213 and S215;
- putative RNA-binding site, a positively charged groove: K41, K28, R57 and R84;
- SAM-binding site, where SAM is stabilized mainly by hydrogen-bonding and van der Waals interactions: S56, G86, W87, K105, H110, D131, V132, D146 and I147; and
- K-D-K-E tetrad from catalytic site: K61, D146, K182 and E218.

Duan et al. [76] aligned the primary sequence of ZIKV MTase to 73 flavivirus sequences and noticed that the most conserved residues are those located in the substrate binding sites cited above (Figure 4D).

Many flavivirus MTase inhibitors candidates were recently discovered. RNA-cap and SAM/SAH analogues are being designed as well as compounds that binds to allosteric sites [73,77]. Good examples of those studies were presented by Jain *et al.* [75]. They developed a SAM analog compound that intrudes the RNA cap-binding site through substitution of the original SAM methyl group by a 4-fluorophenyl moiety. Benmansour *et al*. [78] showed that a DENV MTase allosteric inhibitor, designed by structure-based approach, was also effective against ZIKV. What all these studies, and many others not mentioned here, have in common is the usage of a structure-based drug discovery approach based on X-ray crystallography together with high throughput screening (HTS) techniques, as fluorescence-based thermal shift assays [79], isothermal titration calorimetry (ITC) [80], surface plasmon resonance (SPR) [81], and computational approaches. Up to now, no indication of the NMR assignment of ZIKV or other flavivirus MTase was presented. Nevertheless, as soon as it is available, NMR spectroscopy could also play a major role in structure-based drug discovery of flavivirus MTase inhibitors as exemplified in the NS2B-NS3 protease (Figure 3G). 

### NS5 RdRp domain

NS5 RdRp domains carry out the *de nov*o RNA synthesis of one negative polar-sense RNA from a positive template. The negative polar-sense RNA is then used for the synthesis of positive polar-sense RNA, that later can either be used for protein translation or packed in to form infectious virus particles. DENV NS5 can interact with viral NS3 and with several host proteins as β-importin [58,82,83,84]. Additionally, NS5 behaves as antagonist in host interferon response [76,85]. With this variety of functions, NS5 RdRp domain is for sure a very important target for structure-based antiviral discovery against ZIKV and other flaviviruses. 

Structures of the full ZIKV NS5 and of the NS5 RdRp have been recently published [17,71,76,86]. They have provided crucial information that allowed the comparison of ZIKV NS5 RdRp to those from other flaviviruses. ZIKV RdRp domain is structurally conserved among flaviviruses and adopts right-hand-shaped structure (Figure 4B). Its structure can be decomposed into three subdomains: fingers, residues 321-488 and 542-608; thumb, residues 715-903; and PALM, residues 489-541 and 609-714. The catalytic site is located at the intersection of two tunnels between the fingers and thumb domains. As in other flavivirus, two aspartic acids of the catalytic sites and two Zn^+2^ ions are fundamental for the nucleotidyl transfer catalyze carried out by NS5 RdRp. In the ZIKV, these two residues are located at positions 535 and 665 and the Zn^+2^ ions are coordinated by residues G439, H443, C448 and C451, of the fingers subdomain, and H714, C730, C849, of the thumb subdomain [86]. Another important region characterized by crystal structures of ZIKV NS5 RdRp is the priming loop, formed by residues V785-D810, which is responsible for allosteric positioning of the 3’ terminal of the RNA template in the catalytic site. The nuclear localization region (NLS region), that is composed by βNLS (residues 316-367) and α/βNLS (residues 368-415), is also located in the NS5 RdRp domain. In DENV NS5 RdRp, this region mediates interactions with viral NS3 and with several host proteins [58,82,83,84]. Duan *et al.* [76] compared the ZIKV NS5 RdRp sequence with 73 different flaviviral sequences and identified that the most conserved regions are the priming loop, the RNA tunnel and the Zn^2+^ pocket sites.

As in the case of MTase domain, NS5 RdRp structure-based inhibitor discovery studies, based on X-ray crystallography, have provided encouraging results. Traditionally, two sites of flaviviral RdRp domain are used as inhibitor candidate targets: RNA entry tunnel; and the so called “N pocket”, located in the thumb subdomain, close to the enzyme active site [76,87,88,89,90]. Noble *et al*. [89] obtained a high-resolution crystal structure of the complex of DENV RdRp with the compound NITD107 that binds to RNA template tunnel, avoiding the *de novo* RNA synthesis, and using fragment-based screening via X-ray crystallography, they identified many DENV RdRp inhibitors targeting the “N pocket” [88]. Duan *et al.* [76] found out that almost all residues of the “N pocket” of the DENV RdRp are sequentially and positionally conserved in ZIKV RdRp structure. These results are good examples of the contribution of structure biology of flavivirus to the antiviral discovery efforts. 

## Conclusion and perspectives

As ZIKV spread around the world, the structure biology community combined efforts to obtain the high-resolution structures of ZIKV proteins by using state-of-art methodologies such as X-ray crystallography, biomolecular NMR spectroscopy (NMR) and cryoelectronic microscopy (CryoEM). In a short period of time, these efforts provided important structural information of ZIKV proteins. 

CryoEM provided near-atomic resolution structures of mature, unmatured, free, and antibody-bound ZIKV, contributing immensely to the understanding of ZIKV infection and helping to compare ZIKV to other further studied flaviviruses, as DENV. A good example of this is the higher stability of ZIKV virus particle [33,34], which could be associated with its resistance in a variety of environments as found in urine, semen, breast milk, and saliva [35,36,37,38]. Something that has not yet been characterized by CryoEM is the ZIKV nucleocapsid structure, which demands further developments in experimental and data treatment methodologies. 

X-ray crystallography was historically the most employed technique to characterize virus and viral proteins structures. For ZIKV, this is not different. Structure of six out of ten viral proteins were already obtained by X-ray crystallography (protein E, protein C, NS1, NS2B-NS3, NS3 helicase and NS5). This set of structures includes free, substrate-bound, substrate analog inhibitor-bound and antibody-bound proteins. Highly contributing to structure-based drug discovering and vaccine developing against ZIKV. X-ray crystallography is being extensively used in fragment-based drug development associated to HTS techniques and computational approaches. 

NMR spectroscopy allows structural and dynamical characterization of proteins in atomic scale. Example of this is the application of NMR spectroscopy to characterize the conformational equilibrium of NS2B-NS3 protease, consistently direct towards a closed state, by NMR relaxation experiments. NMR spectroscopy was also used to map substrate and inhibitor candidates binding sites by titration experiments. NMR and computational techniques can also play an important role in ZIKV protein E conformation epitope identification, as carried out by Moraes *et al.* [52] for DENV. Another important contribution of NMR can arise from its capacity to characterize very dynamic proteins or flexible regions within proteins, as in cases in which X-ray crystallography has some limitations. 

Nowadays, state-of-art experiment techniques for protein structure determination as CryoEM, X-ray crystallography and NMR spectroscopy are providing complementary structural and dynamical information for a great number of proteins and macromolecular systems as viruses. Combined to other experimental and computational methodologies, they are contributing to deepen the understanding of ZIKV infection, replication and blockade. Experimental and theoretical improvements will, together with new protocols for sample production, certainly accelerate the ZIKV structure characterization and help to answer important remained questions. 

### Abbreviations

ATP: adenosine triphosphate; CryoEM: cryogenic electron microscopy; DENV: dengue virus; DI: domain I of protein E; DII: domain II of protein E; DIII: domain III of protein E; ER: endoplasm reticulum; Fab: fragment antigen-binding; GTP: guanosine triphosphate; HCV: hepatitis C virus; HTS: high throughput screening; ITC: isothermal titration calorimetry; JEV: Japanese encephalitis virus; KUNV: Kunjin virus; MD: molecular docking; MTase: methyltransferase; MVEV: Murray Valley encephalitis virus; NMR: nuclear magnetic resonance; NS1: non-structural protein 1; NS2: non-structural protein 2; NS3: non-structural protein 3; NS4: non-structural protein 4; NS5: non-structural protein 5; Protein C: capsid protein C; Protein E: glycoprotein E; Protein M: membrane protein M; RC: replication complex; RdRp: RNA-depended RNA polymerase; RNA: ribonucleic acid; SAH: S-adenosyl-L-homocysteine; SAM: S-adenosyl-L-methionine; SAXS: small angle X-ray scattering; scFv: single-chain variable fragment; SPR: surface plasmon resonance; WNV: West Nile virus; YFV: yellow fever virus; ZIKV: Zika virus.
